# Disparities in Industry Payments Between Orthopaedic Surgery and General Surgery Residents

**DOI:** 10.2106/JBJS.OA.26.00029

**Published:** 2026-07-24

**Authors:** Cemil C. Gedik, Claire Saltzman, Parker D. Boyce, Jonathan R. Dubin, An-Lin Cheng, Houssam Bouloussa, Akin Cil

**Affiliations:** 1 Department of Orthopedic Surgery, University of Missouri—Kansas City, School of Medicine, Kansas City, Missouri; 2 University of Missouri—Kansas City, School of Medicine, Kansas City, Missouri; 3 Department of Biomedical and Health Informatics, University of Missouri—Kansas City, Kansas City, Missouri

## Abstract

**Background:**

Industry funding may support surgical training but also raises conflict-of-interest concerns, particularly among financially vulnerable residents. This study examined whether the prevalence and magnitude of Centers for Medicare & Medicaid Services (CMS) Open Payments general payments differed between postgraduate year 5 (PGY-5) orthopaedic surgery (OS) and general surgery (GS) residents graduating in 2025, and whether these payments varied by institution type and geographic region.

**Methods:**

We performed a cross-sectional analysis of CMS Open Payments (2018-2024) for PGY-5 residents in OS and GS graduating in 2025. Residency programs were identified, and PGY-5 rosters were compiled from program websites. Residents were linked to Open Payments records. Payments were categorized as Consulting Fees/Grants/Charity, Travel/Education Support, and Hospitality. Payment prevalence and payment amounts were compared between specialties, with adjustment for institution type and geographic region.

**Results:**

Across 150 institutions (120 GS programs, 117 OS programs), 1,591 residents were analyzed. Overall, 861 of 1,591 residents (54%) had ≥1 payment; OS residents had higher prevalence than GS (57% vs. 52%; OR 1.25, 95% confidence interval [CI]: 1.03-1.53; p = 0.03). Unadjusted mean payments were $3,176 (OS) versus $798 (GS) (p < 0.0001). Adjusted mean payments remained higher for OS ($3,202) than GS ($653), a 4.90-fold difference (p < 0.0001), with significant interactions by institution type and region.

**Conclusions:**

Over half of PGY-5 GS and OS residents receive industry payments, although OS receive substantially more overall. These payments are highly concentrated at the top tier of recipients. Training programs, governing bodies, and other key stakeholders should consider developing a structured approach that capitalizes on the industry financial support while minimizing bias in resident education and delivering a more equitable distribution to all residents.

**Level of Evidence or Clinical Relevance:**

Level III (cross-sectional observational study); clinically relevant to graduate medical education policy and conflict-of-interest oversight. See Instructions for Authors for a complete description of levels of evidence.

## Background

Industry support has long played a role in resident education, particularly in procedural fields where funding may help support skills laboratories, courses, and technical training^1^. However, these relationships remain controversial because of the potential for conflicts of interest^2,3^. Between 2013 and 2022, drug and device companies paid nearly $12 billion to more than 800,000 physicians in the United States^4^. Residents may be especially susceptible to these influences because they often carry substantial educational debt while earning relatively modest salaries^5,6^.

The Physician Payments Sunshine Act was enacted to improve transparency in physician-industry relationships, and since 2014, the Centers for Medicare & Medicaid Services (CMS) Open Payments database has publicly reported many forms of nonresearch payments^7,8^. Prior analyses have shown that surgeons, particularly orthopaedic surgeons, are among the highest recipients of industry payments, with payments highly concentrated among a small proportion of physicians^4,9-11^. An analysis of the first 5 years of Open Payments Database revealed that the top 1% of orthopaedic surgeons received $265.8 million, accounting for 58% of all industry payments in the specialty^12^. Although attending-level data are well described, less is known about how this relationship extends to residents.

The ACGME recognizes that navigating industry relationships is part of professionalism and systems-based practice. Prior studies suggest that industry-sponsored activities are common in surgical training^1,9,13^. The objectives of this study were to (1) quantify and compare resident exposure to reportable industry payments during training between orthopaedic surgery and general surgery residents in the 2025 graduating class and (2) examine how this exposure varied by institutional type and geographic region.

## Materials and Methods

We conducted a cross-sectional analysis of industry payments recorded in the CMS Open Payments program for postgraduate year 5 (PGY-5) residents in orthopaedic surgery (OS) and general surgery (GS) graduating in 2025. GS was selected as the comparator because it shares the same 5-year training structure as OS and similarly involves highly technical, skills-based training, allowing direct comparison at the PGY-5 level.

**Fig. 1:**
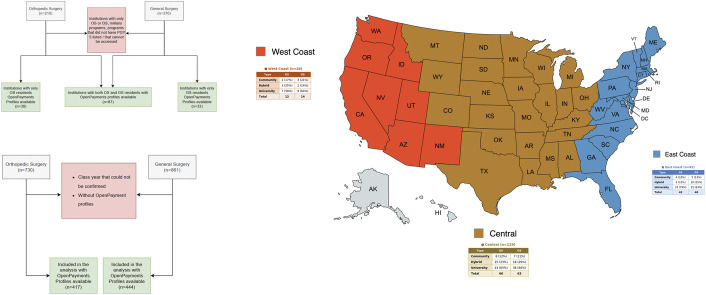
Flowchart for included institutions and residents for general surgery and orthopaedic surgery programs by institution type and region.

Residency programs were identified using the AMA FREIDA database^14^. Programs were classified by geographic region (East Coast, Central, West Coast) according to location and by institution type (University, Community, or Hybrid) according to primary affiliation and teaching status. Hybrid programs were defined as those in which residents train across both community and university hospital settings, regardless of the primary employing institution. All institutions offering GS and/or OS residency training were included, yielding 150 unique institutions (120 GS programs and 117 OS programs; Fig. 1). Program distributions by institution type and geographic region are also shown in Fig. 1

PGY-5 rosters for the 2025 graduating class were assembled from program websites and archived pages. Resident-level payments were extracted from the CMS Open Payments files (2018-2024 data). Residents were linked to Open Payments records by Covered Recipient Profile ID or NPI.

For each resident on CMS Open Payments database, we determined the presence of any reported industry payment during the study period and quantified the total payment amount when present. Payments were manually categorized into the following: Consulting Fees/Grants/Charity (Grants), Travel/Education Support (Other), and Hospitality (food/beverage).

Chi-square tests were used to compare the proportion of residents receiving industry payments between specialties. Odds ratios with 95% confidence intervals (CIs) were calculated. Statistical significance was set at p < 0.05. Analyses were performed using Python (v3.1) with pandas and scipy libraries and SAS v9.4 (SAS Institute Inc). Visualizations were made with Python and React.

We compared industry payment amounts across specialties, institution types, and geographic regions using multivariable regression models. Analyses were conducted at 3 levels: (1) aggregate totals by program, (2) individual resident totals, and (3) individual transactions. Models included Specialty (Orthopaedics vs. General Surgery), Institution Type (University, Community, Hybrid), and Geographic Class (East, Central, West), along with their interactions. Because payment amounts were right-skewed (i.e., most payments were small, but some were very large, producing a long upper tail in the distribution), we log-transformed the data for analysis and present results as adjusted mean payments (back-transformed to dollars) with 95% confidence intervals. Multiple comparisons were adjusted using the Tukey-Kramer method to reduce Type I error.

## Results

The average number of residents per program was similar between specialties: 3.72 ± 1.90 (range 1-9) for General Surgery and 3.61 ± 1.58 (range 1-8) for Orthopaedics. Among the 1,591 residents analyzed, industry payments were reported for 861 residents (54%) overall, with significant variation by specialty. Orthopaedics residents had a higher percentage of industry payments (417/730, 57%) compared with GS residents (444/861, 52%; χ^2^ = 4.69, p = 0.03, OR = 1.25, 95% CI: 1.03-1.53). We present both unadjusted and adjusted results: Unadjusted means provide a straightforward description of the raw payment differences, while adjusted means account for confounding by institution type and geography, and facilitates interpretation of potential skewness of data.

### Orthopaedic Surgery Compared With General Surgery: Resident-Level Analysis

CMS database showed that OS residents received substantially higher industry payments than GS residents. In unadjusted analyses, mean payments were $3,176.46 (95% CI: $2,867.40–$3,485.53) for Orthopaedics vs. $797.99 (95% CI: $672.13–$923.85) for GS, representing a 3.98-fold difference (p < 0.0001). After adjustment for institution type and geographic region, the mean payment was $3,201.77 (95% CI: $3,193.95-$3,209.62) for orthopaedic residents versus $652.69 (95% CI: $648.91-$656.49) for general surgery residents (adjusted p < 0.0001).

The specialty disparity varied significantly by institution type (interaction p < 0.0001, Table I).

**TABLE I:** Magnitude of Difference by Specialty and Institution Type

Institution Type	OS Adjusted Mean (95% CI)	GS Adjusted Mean (95% CI)	Fold Difference
Hybrid	$3,987.26 ($3,973.15–$4,001.42)	$533.85 ($528.71–$539.05)	7.47
Community	$2,985.80 ($2,969.00–$3,001.79)	$628.80 ($621.86–$635.82)	4.75
University	$2,757.00 ($2,750.29–$2,763.73)	$828.29 ($824.07–$832.54)	3.33

CI = confidence interval, GS = general surgery, and OS = orthopaedic surgery.

### Geographic Location

**Fig. 2:**
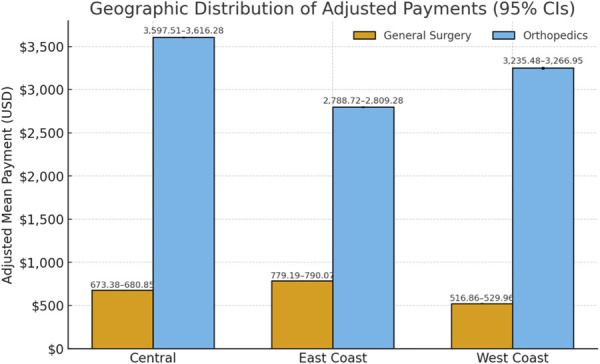
Adjusted mean industry payments per resident by region for general surgery and orthopaedics. Bars show adjusted means (USD). Numerical 95% CI limits are labeled above each bar. CI = confidence interval.

Overall adjusted mean payments differed significantly by region (*p* < 0.0001), with the Central region showing the highest payments (mean $1,562.76), followed by the East Coast (mean $1,481.93) and West Coast (mean $1,304.44). The specialty-geography interaction was statistically significant (*p* < 0.0001). Comparison of GS and OS for geographic location is shown in Fig. 2.

### Payment Concentration and Inequality

**Fig. 3:**
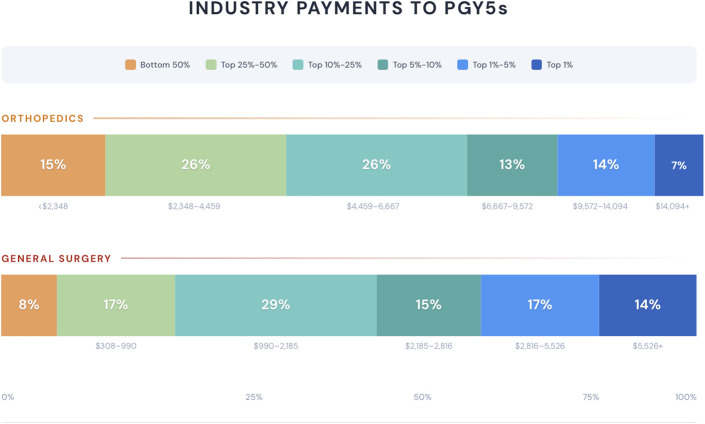
Distribution of industry payments to PGY5 residents by specialty and income group. PGY5 = postgraduate year 5.

When all PGY5 residents were ranked by total industry payments, orthopaedics residents comprised 93% of the top payment decile (80/86), whereas GS residents comprised 7% (6/86; χ^2^ = 76.07, p < 0.0001, Phi coefficient = 0.297). Within each specialty, 80 of 417 orthopaedics residents (19%) exceeded the overall top-decile payment threshold, compared with only 6 of 444 general surgery residents (1%), a 14.2-fold difference in the likelihood of reaching the highest payment tier (Fig. 3).

In general surgery, the top 1% of residents (n = 5) received 14% of total payments and the top 10% (n = 45) received 46%, whereas the bottom 50% (n = 222) received only 8% (mean, $129.66 per resident; range: $11.79–$305.00). In OS, the top 1% (n = 5) received 7% of total payments and the top 10% (n = 42) received 33%, whereas the bottom 50% (n = 208) received 15% (mean, $966.40 per resident; range: $14.72-$2,318.24).

### Payment Type (Per Transaction)

In a sensitivity analysis classifying each payment into Grant, Hospitality, and Other categories, the Specialty × Payment Class interaction remained highly significant (F-statistic-ANOVA = 10.43, p < 0.0001). When looking at residents receiving grants, GS residents received significantly higher adjusted mean payments in the Grant category ($4,651.18, 95% CI: $1,620.38–$13,351) compared with OS residents ($2,346.20, 95% CI: $1,252.69–$4,394.28), representing a nearly 2-fold difference. Conversely, OS residents received higher Other payments ($488.11, 95% CI: $455.82-$522.68) compared with GS ($413.39, 95% CI: $369.25-$462.81), while hospitality payments showed minimal variation between specialties (GS: $64.69, 95% CI: $61.35-$68.20; OS: $59.03, 95% CI: $56.60-$61.56).

At the payment count level, OS residents received more hospitality payments (mean 24.30, SD 22.60, range 0-120) compared with GS residents (mean 6.03, SD 7.46, range 0-53), with a 4.0-fold difference. This disparity was equally pronounced for other payments, where orthopaedics averaged 3.56 payments (SD 4.60, range 0-30) versus 0.88 (SD 2.07, range 0-21) for GS. Overall, there were only 25 grant payments total going to 18 OS and 4 GS residents, with 2 OS and 1 GS receiving 2 grants.

## Discussion

This cross-sectional analysis reveals that while the proportion of residents receiving any industry payment was broadly comparable between the specialties, with just over half of residents within each specialty reporting payments during their training, the number and magnitude of payments differed substantially. OS residents received four-times the hospitality and other payments during training, yielding a 5-fold greater total dollar value in industry payment, after adjustment for institution type and geography.

Almaguer et al. analyzed 2014 to 2016 data for OS residents and reported that 70% of PGY-5 residents received industry payments, with a mean of $4,070 among recipients^15^. Hogan et al. examined 65,992 residents across 6 specialties (OS, GS, urology, internal medicine, obstetrics and gynecology, family medicine) during the 2020 to 2021 academic year and found that 39% of OS residents received payments (“gifts”) with a median per-recipient value of $526^13^. This analysis occurred during the COVID-19 pandemic, likely suppressing payment volumes^13,16^. The consistency of orthopaedic surgery's outsized industry engagement across studies spanning a decade suggests that this pattern reflects structural features of the specialty.

Prior work from Almaguer et al. showed education and grants comprised over 60% of orthopaedic resident payments from 2014 to 2016, with travel and food accounting for most of the remainder while Hogan et al. reported that food and beverage payments constituted the vast majority of resident payments^13,15^. Nakayama and Bozeman similarly found 58% of surgery program directors reported industry-paid guest lecturers and 64% reported industry-sponsored courses as appropriate educational activities^1^. The extent to which core surgical education has become reliant, or potentially dependent, on industry-sponsored activities warrants scrutiny.

While OS residents received higher total payments overall, GS residents received higher grant payments (adjusted mean $4,651 vs. $2,346). However, grant-related payments represented only a small proportion of payments with only 25 total grants going to 22 residents; therefore, this finding should be interpreted cautiously. Comparable patterns are described in internal medicine training, where most pharmaceutical industry support takes the form of food for conferences (91%) and educational materials (83%), underscoring a broader culture of normalized industry participation in graduate medical education^17^.

Our findings on payment concentration parallel those reported at the attending level. OS residents constituted 93% of the highest payment recipients and were 14 times more likely than GS residents to fall into the top decile of payments. This mirrors the inequality described by Samuel et al., who reported a Gini coefficient (a measure of inequality ranging from 0, indicating perfectly equal distribution, to 1, indicating maximum concentration) of 0.956 among orthopaedic surgeons, with the top 10% receiving 95% of all industry payments^11^.

Prior research has linked sponsoring institution ownership to resident payment acceptance, with for-profit institutions showing 3.5-fold higher rates than federal institutions^13^. Pope et al. further demonstrated that physicians at teaching hospitals accepting higher-value payments tend themselves to receive higher-value payments^18^. Our analysis reveals these institutional effects are seen in orthopaedics, suggesting an interaction between specialty culture and institutional factors. Hybrid institutions demonstrated the largest payment disparity (7.47-fold) between specialties. This pattern implies institutional culture, and local industry relationships interact with specialty norms to shape resident exposure.

Several limitations should be acknowledged. First, payments to resident physicians are not required to be reported under the Sunshine Act, and several categories of lower-value or exempt transfers are not captured. Furthermore, we did not include payments made directly to training programs or program directors. Second, this cross-sectional analysis evaluates a single graduating cohort and cannot assess temporal trends or long-term impact. Third, resident identification was performed manually using program websites, which may incompletely capture trainees, and preclude collection of demographic data. Fourth, the analytic sample included 150 unique institutions from 210 OS and 370 GS programs initially identified. This attrition primarily reflected inability to identify PGY-5 rosters from program websites and introduces the possibility of selection bias if excluded programs differed systematically from those included.

Finally, this study quantifies exposure but does not measure behavioral outcomes associated with that exposure. However, there is literature connecting behavioral bias in physicians receiving industry payments or even gifts as seemingly innocuous as a pen^19-21^.

The federal government spends over $15 billion annually on resident education or about $120,000 per resident^22,23^. The true annual cost to train a resident is estimated at approximately $150,000^24,25^. Industry funding may offset some of this gap; however, our data show the money being highly concentrated at the upper echelons of recipients and not distributed equitably among learners. Furthermore, the sizable proportion of hospitality funding implies money is spent often on activities tangential to learning. Last, GS residents enjoyed substantially less industry support than OS residents, and while not specifically investigated in our study, there is no reason to suspect GS residents receive inferior training or surgical preparedness. This questions whether industry funding provided to OS residents is necessary or just more accessible.

Regardless of whether industry funding is necessary or not, its prevalence in orthopaedic resident education mandates the consideration of a structured approach by training programs, governing bodies, and key stakeholders to minimize the effects of biased learning. For example, financial support from industry partners could be given to third-party entities, such as professional societies, which can then distribute the funding more equitably and with less risk of direct industry influence. Already, this is common, with multiple professional societies offering scholarships, sponsored by industry partners, for residents to attend educational courses^26-28^.

## Conclusion

Industry payments to OS and GS residents is common, but OS residents receive more frequent, higher-value, and more concentrated payments than GS residents. These findings suggest strong potential for bias in the education of future surgeons. Training programs, governing bodies, and key stakeholders should consider a structured and strategic approach that capitalizes on the willingness of industry partners to provide financial support for resident education while minimizing bias and delivering a more equitable distribution to all residents.

## Funding

The authors have no sources of funding to declare for this manuscript.

## Data Availability

Publicly available Open Payments Database was used to obtain data.
